# Lyophilized Matrix Containing Ready-to-Use Primers and Probe Solution for Standardization of Real-Time PCR and RT-qPCR Diagnostics in Virology

**DOI:** 10.3390/v12020159

**Published:** 2020-01-30

**Authors:** Laurence Thirion, Audrey Dubot-Peres, Laura Pezzi, Iban Corcostegui, Mhammed Touinssi, Xavier de Lamballerie, Remi N. Charrel

**Affiliations:** 1 Unité des Virus Emergents (UVE: Aix Marseille Univ, IRD 190, INSERM 1207, IHU Méditerranée Infection), 13005 Marseille, France; laurence.thirion@ird.fr (L.T.); audrey.dubot@ird.fr (A.D.-P.); laura.pezzi3@studio.unibo.it (L.P.); iban.corcostegui@gmail.com (I.C.); mhammed.touinssi@efs.sante.fr (M.T.); xavier.de-lamballerie@univ-amu.fr (X.d.L.); 2 EA7310, Laboratoire de Virologie, Université de Corse-Inserm, 20250 Corte, France; 3 Etablissement Français du Sang Alpes Méditerranée, 13005 Marseille, France; 4 Emerging Pathogens Institute, University of Florida, Gainesville, FL 32610, USA

**Keywords:** freeze-drying, lyophilization, PCR, diagnosis, virus, pathogen, TaqMan, emerging, epidemic

## Abstract

Real-time molecular techniques have become the reference methods for direct diagnosis of pathogens. The reduction of steps is a key factor in order to decrease the risk of human errors resulting in invalid series and delayed results. We describe here a process of preparation of oligonucleotide primers and hydrolysis probe in a single tube at predefined optimized concentrations that are stabilized via lyophilization (Lyoph-P&P). Lyoph-P&P was compared versus the classic protocol using extemporaneously prepared liquid reagents using (i) sensitivity study, (ii) long-term stability at 4 °C, and (iii) long-term stability at 37 °C mimicking transportation without cold chain. Two previously published molecular assays were selected for this study. They target two emerging viruses that are listed on the blueprint of the WHO as to be considered for preparedness and response actions: chikungunya virus (CHIKV) and Rift Valley fever phlebovirus (RVFV). Results of our study demonstrate that (i) Lyoph-P&P is stable for at least 4 days at 37 °C supporting shipping without the need of cold chain, (ii) Lyoph-P&P rehydrated solution is stable at +4 °C for at least two weeks, (iii) sensitivity observed with Lyoph-P&P is at least equal to, often better than, that observed with liquid formulation, (iv) validation of results observed with low-copy specimens is rendered easier by higher fluorescence level. In conclusion, Lyoph-P&P holds several advantages over extemporaneously preparer liquid formulation that merit to be considered when a novel real-time molecular assay is implemented in a laboratory in charge of routine diagnostic activity.

## 1. Introduction

The detection of the genome of pathogens has become the gold standard technique for direct diagnosis because of excellent sensitivity and specificity, and due to its capacity to provide a result within hours [1,2]. Nonetheless, there are several factors that merit to be mastered in order to obtain results that can be steadily validated. Among those factors, ensuring and maintaining the quality of the components of the reaction mix, in particular oligonucleotide primers and fluorescent probe. It is important to distinguish basic research context from diagnostic context. The latter can hardly suffer delays in result validation. It is important to underline that clinical microbiology laboratories are now frequently grouping assays for viruses, bacteria, fungi, and parasites not only for diagnosis but also for further characterization of pathogen through genotyping or resistance detection. This obviously rapidly leads to a large number of assays to run daily or weekly, and as a consequence a rather large number of potential pitfalls [3]. Although failure of one of the mix components is easily detected when the positive control does not provide adequate results, such a situation has an important impact on the laboratory throughput due to delayed results, reordering reagents, increased laboratory costs, increasing technical workload, and feeling insecure concerning the capacity of biologists to provide results and of clinicians to obtain results timely. Whether this can appear as a minor problem for laboratories using few in-house assays, it can rapidly become hectic when a larger number of in-house assays are used for routine diagnostic purpose. There are several causes for failures linked to primers and/or probes such as light exposure that deteriorates fluorescence of the probe, repeated freeze-thaw cycles resulting in DNA degradation, mistakes in final concentrations, or pipetting errors when the reaction mix is prepared [4]. Such problems have been at least partially solved in commercial kits through serial aliquoting and lyophilization or ambient-temperature stable reagents. Ready-to-use reagents reduce the risk of human errors. Lyophilized reagents are more stable than liquid formulations. The combination of both measures aims at improving the quality of the results. We describe here a process of preparation of oligonucleotide primers and hydrolysis probe in a single tube at predefined optimized concentrations (P&P for Primers and Probe(s)) that are stabilized via lyophilization (Lyoph-P&P). We have compared the performances of two selected assays (Lyoph-P&P vs. the classic protocol using frozen reagents) and have studied the long-term stability of Lyoph-P&P in native and rehydrated formulations. Selected assays target two emerging viruses that are listed on the blueprint of the WHO as to be considered for preparedness and response actions [5]: chikungunya virus (CHIKV), a single-stranded positive-sense RNA alphavirus, and Rift Valley fever phlebovirus (RVFV), a tri-segmented, single-stranded negative-sense RNA phlebovirus.

## 2. Materials and Methods

### 2.1. Experimental Conditions Common to All Assays

Specific hydrolysis probe-based real-time RT-PCR (RT-qPCR) assays were selected for the detection of CHIKV and RVFV viruses (Table 1). Upon reception, lyophilized primers (Eurogentec) and probes (Applied Biosystems, ThermoFisher) were regenerated in Tris-HCl (5 mM pH 8.5) buffer, to obtain (i) a 100 µM stock solution and (ii) a 10 µM working solution which both were stored at −20 °C. Optimal concentrations for primers and probes have been experimentally determined and are indicated in Table 1. RT-qPCR assays used 10 μL of RNA with the Superscript^®^ III Platinium^®^ One-Step Quantitative RT-PCR kit (Invitrogen-ThermoFisher Scientific, Waltham, MA, USA) in a final volume of 30 μL following the manufacturer’s protocol. Reactions were performed on a BioRad real-time thermal cycler CFX96™ and CFX Manager Software version 3.1 following thermal profile: 30 min at 50 °C (1 cycle), 2 min at 95 °C (1 cycle), (15 s at 95°C, 45 s at 60 °C) (45 cycles). The result was considered negative for Ct values ≥ 40.

### 2.2. Synthetic Standard RNA for CHIKV and for RVFV

For each RT-qPCR assay, the target sequence, preceded by the T7 promoter sequence, was inserted into pUC 57 plasmid (Genscript). Each plasmid (4 μg) was regenerated, then 10-fold serially diluted using Tris HCl buffer (5 mM pH 8.5). A range of dilutions was submitted to M13 PCR (Primer 5′-3′: M13F TGT AAA ACG ACG GCC AGT, M13R CAG GAA ACA GCT ATG ACC). The PCR product from the highest plasmid dilutions giving the strongest amplification band on agarose gel was selected for subsequent transcription. Synthetic RNA transcripts were synthesized from 8 μL of PCR product using MEGAshortscript™ T7 Transcription Kit (Ambion™) following the manufacturer’s instructions. Plasmid DNA was removed with DNase (Turbo DNA-Free™, Invitrogen™), then, the RNA transcript was purified using the Monarch^®^ PCR & DNA Cleanup Kit (Biolab) following manufacturer’s instructions. RNA concentration (copy number per µL) was calculated for each standard from RNA concentration measured using NanoDrop^®^ 1000 from Thermo Scientific. The standard concentrations were 8.8 × 10^11^ RNA copies/µL for CHIKV and 7.7 × 10^11^ RNA copies/µL for RVFV. Each standard was serially diluted using AVE buffer-RNA carrier (10 ng/µL, Qiagen), then each dilution was aliquoted and stored at −80 °C until use (AVE buffer is the name provided by Qiagen and contains RNase-free water with 0.04% sodium azide). The quality of each standard was checked by submitting serial dilutions to RT-qPCR (as described above) and to qPCR (using LightCycler^®^ DNA Master HybProbe, Roche). A difference in the limit of detection (LOD) of at least 9 log between RT-qPCR and qPCR, corresponding to negligible traces of remaining DNA not interfering with RNA quantification, was considered acceptable.

### 2.3. Preparation of P&P Liquid Solution before Lyophilization

The concentration of the different components of the P&P can be slightly increased (lyophilization factor) from the ones used for extemporary preparation; adequate concentrations are listed in Table 2. The corresponding volume of P&P solution was dispensed in 2-mL glass vials (WHEATON^®^, Dominique Dutscher, Brumath, France). Two µL of sucrose 1 M used as a stabilizing agent and, optionally, 1.5 µL of red food coloring E222 (1/600 in water) was added in order to visualize better dispensing of the P&P. The volume was adjusted to 200 µL using molecular grade water (UltraPureTM Distilled Water, Invitrogen). In order to prevent insufficient volume for the ultimate reactions due to pipetting errors, a safety margin was included in the calculation; for instance, to prepare a 16-test vial, volumes are calculated for 18 tests. Glass vials containing P&P solution to perform 8 to 96 reactions can be prepared using the protocols presented in Table 2.

### 2.4. Lyophilization Protocol

Glass vials containing P&P liquid solution (as prepared in Table 2) were stored at −40 °C for at least 2 h prior lyophilization. Frozen vials were lyophilized in a Pilot bench freeze-dryer (Cryotec, France) using the program#1: 1.208 bar at −20 °C for 15 min, 0.708 bar at −20 °C for 120 min, 0.708 bar at −10 °C for 60 min, and 0.402 bar at −10 °C for 120 min; thereafter, program#2 was launched and consisted of 0.402 bar at −20 °C for 60 min and 0.231 bar at −20 °C for 120 min. Upon completion, vacuum was broken by injection of nitrogen gas (AirProducts); then, the vials were sealed and stored at −20 °C. All vials used for this study come from the same drying process.

### 2.5. Rehydration of Lyoph-P&P before Use

P&P vials containing 16 tests were regenerated with 79.2 μL of AE Elution Buffer (Macherey-Nagel) and homogenized by multiple pipetting of a 50 µL-volume at least 10 times in the vials; then, rehydrated P&P was incubated at room temperature for 10 min, after which 10 times multiple pipetting was done again; these steps are critical to ensure adequate homogenization (Table 3).

### 2.6. Stability at 4 °C after Regeneration of Lyoph-P&P

After regeneration, rehydrated P&P were stored at 4 °C for 2 weeks. At three time points (day 0, day 7, and day 14), RT-qPCR was performed using extemporaneously prepared liquid P&P. RT-qPCR was performed on three replicates for each of three dilutions (10^−8^, 10^−7^, and 10^−6^) of corresponding RNA (Table 4).

### 2.7. Stability of Lyoph-P&P at 37°C for Shipping Mimicry

Lyoph-P&P vials (not rehydrated) were placed at 37 °C for 2 weeks to simulate conditions that might be encountered during oversea shipping with failure of the cold chain or shipping without cold chain conditions. Vials were rehydrated at day 2, day 4, and day 7, and results were compared with those observed at day 0 using three replicates.

### 2.8. Analytical Sensitivity

The measure and comparison of the analytical sensitivity of CHIKV and RVFV assays were done by using synthetic standard RNAs. Serial five-fold dilutions of the quantitated RNAs were prepared using AVE buffer-RNA carrier (Qiagen). Six decreasing concentrations (1.00 × 10^−8^, 8.00 × 10^−11^, 1.60 × 10^−11^, 3.20 × 10^−12^, 6.40 × 10^−13^, and 1.28 × 10^−13^) were tested using three replicates for each. A Ct ≥ 40 was considered as negative. LOD is considered as the lowest amount of analyte in a sample that can be detected with (stated) probability, although perhaps not quantified as an exact value. In this study the LOD was defined as the number of RNA copies/µL contained in the highest dilution for which the three replicates were positive. The LOD 95 is the analyte concentration that produces at least 95% of positive replicates.

### 2.9. Clinical Samples

A total of 70 clinical samples that were tested positive for CHIKV RNA at the National Reference Centre for Arboviruses were kindly provided by her Director (Dr. Isabelle Leparc-Goffart) to be re-tested comparatively using the extemporaneously prepared liquid formulation of mix and using the Lyoph-P&P as described in this study.

## 3. Results

### 3.1. Decscription of the Lyoph-P&P Method

**Figure 1:**
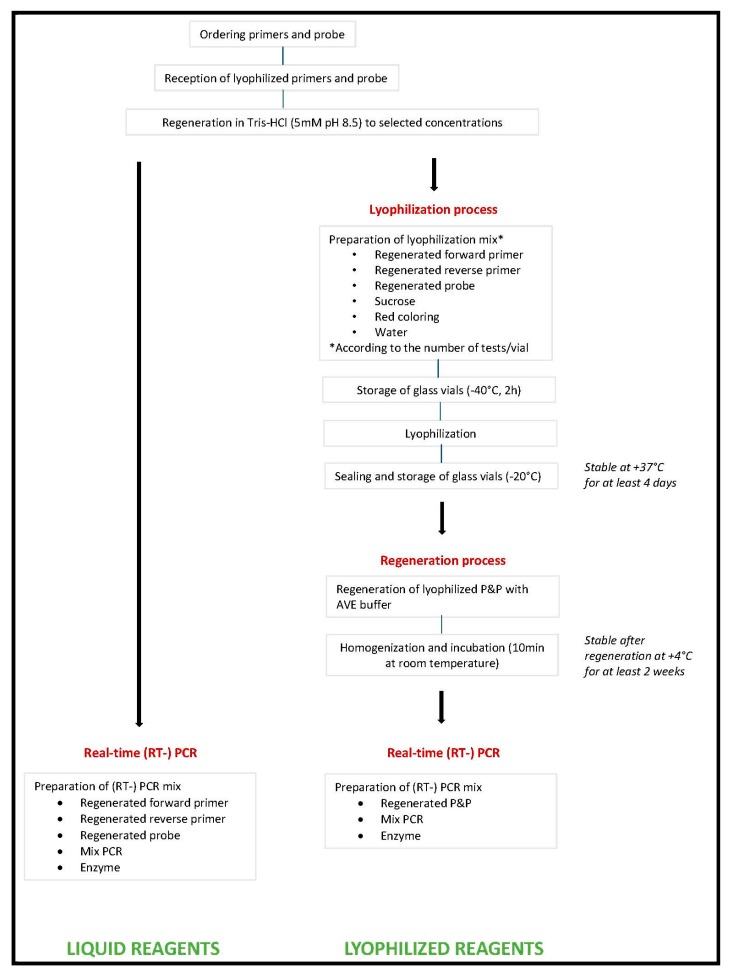
Schematic representation of the two formulas tested in this study.

**Figure 2:**
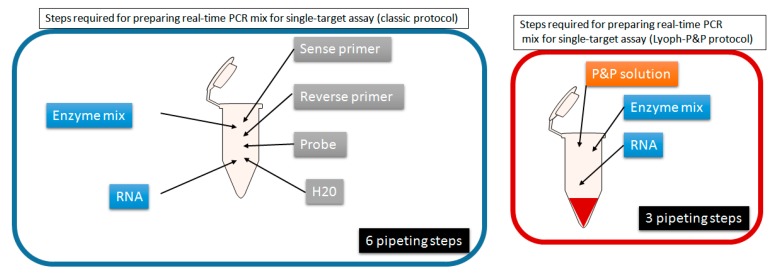
PCR/RT-PCR mix preparation using the extemporaneous formulation *versus* Lyoph-P&P method.

Differences between the Lyoph-P&P method compared with the traditional extemporaneous preparation is illustration in Figure 1; Figure 2.

### 3.2. Stability at 4 °C after Rehydration of Lyoph-P&P

The results are presented in Table 5. For CHIKV, results observed with the Lyoph-P&P were systematically better than those obtained when using the liquid formulation extemporaneously prepared. For RVFV, the results observed with the Lyoph-P&P were almost identical to those obtained with the liquid formulation; Lyoph-P&P Ct values were never higher than 1.10 Ct (as compared to the liquid reference), corresponding to a theoretical difference of 1/2 log.

### 3.3. Stability of Lyoph-P&P at 37°C for Shipping Mimicry

For CHIKV, results observed after maintaining the Lyoph-P&P at 37 °C to mimic shipping conditions without cold chain or degraded conditions were similar, although slightly better, at days 2, 4, and 7 compared with other conditions suggesting that the stability of freeze-dried reagents was excellent. For RVFV, the lowest Ct values were observed at days 0, day 2, and day 2 for 7700, 770, and 77 RNA copies/µL, respectively; however, Ct values observed between day 0 and day 4 were very similar suggesting that degraded shipping conditions might affect in a very limited manner the quality of the Lyoph-P&P (Table 6).

### 3.4. Analytical Sensitivity

The results are presented in Table 7. For CHIKV, the samples containing 14 RNA copies/µL were detected with both the liquid and the Lyoph-P&P reagents. In contrast, none of the three samples containing three RNA copies/µL was found positive using the liquid reagents, whereas all three samples were found positive using the Lyoph-P&P; moreover, all three samples containing 0.56 RNA copies/µL were also found positive with the Lyoph-P&P. This denotes a better analytical sensitivity of the Lyoph-P&P compared with the liquid formulation for the detection of CHIKV RNA.

For RVFV, the samples containing 12 RNA copies/µL were detected with both the liquid and the Lyoph-P&P reagents. In contrast, only one out of three samples containing two RNA copies/µL was found positive using the liquid reagents, whereas all three samples were found positive using the Lyoph-P&P. This denotes a better sensitivity of the Lyoph-P&P compared with the liquid formulation for the detection of RVFV RNA.

### 3.5. Comparative Analysis of Sensitivity on CHIKV RNA Positive Clinical Samples

As shown in Table 8, mean Ct values and SD observed using the Lyoph-P&P were lower than those obtained with the extemporaneously prepared liquid formulation in 65/70 and 41/70 samples, respectively.

## 4. Discussion

Real-time molecular techniques are now the reference methods for the direct diagnosis of pathogens. Increasingly, automation has developed in order to reduce the number of steps prone to human errors, and now the tendency is towards random access tests where all steps are automated until biological validation. However, this approach, developed by diagnostics companies such as Hologic, Roche, Abbott, Cepheid, BioMerieux among others, focus on marketable tests meaning that a certain amount of assays has to be expected in the business plan before such assays are developed. Commercially developed assays need to be registered by regulation agencies before they are available on the market; in many cases, this leads to delays that are not compatible with preparedness and response activities, as witnessed by the current situation with the novel coronavirus. Moreover, often the development and licensing of a novel assay is conditioned by the size and volume of anticipated future market which is not necessarily considered as profitable. Lastly, the price for such assay is almost always not compatible with daily use in laboratories of developing countries.

Obviously, a large number of microbial targets will never be addressed by such random access technologies due to their lack of marketability although they might be major human, veterinary, or plant pathogens. It is worrisome that this situation is contradictory with the principle of preparedness and response to emerging pathogens [5].

Although real-time molecular techniques are now implemented worldwide, laboratories still face technical problems due to the large number of parameters and reagents to manage, the stability of respective reagents, and the multiple steps from patient to result [7]. Among the parameters to consider in the process of clinical diagnostics, primers and probe are among those that require the largest number of steps to operate from the stage of ordering the reagents (primers and probe(s)) to the launching the PCR or RT-PCR reaction onto the thermal cycler. Even in the simplest format, two oligonucleotide primers and one probe are ordered from manufacturing companies. Upon reception, each of these three tubes has to be rehydrated and/ or diluted to prepare a stock solution (usually 100 µM) and a working solution (usually 10 µM), both stored at −20 °C for stability. Then for each experiment, specific volumes of each of the three working solutions have to be manipulated to prepare the PCR mix solution which is then distributed into individual reaction tubes or plates. In contrast, the enzyme mix is now mostly commercialized in a 2X solution which requires to perform few steps until distribution into the reaction tubes. Last the “to be tested” solution of total nucleic acids, RNA, or DNA is distributed. Since the manipulation of primers and probe requires the largest number of steps, we selected it as target for simplification (Figure 1 and Figure 2).

The aim was (i) to produce a ready-to-use Primers & Probe mix (P&P) for each pathogen to be tested, (ii) to validate the resulting P&P in its Lyophilized form (Lyoph-P&P), (iii) to optimize the whole process and to make it available and usable easily to laboratories willing to adopt the same approach. The ultimate objective was to produce reagents amenable to any laboratory having the capacity to perform real-time molecular detection of pathogens for diagnostic purpose.

The assays that were selected for comparative evaluation in this study have been thoroughly evaluated for the respective detection of CHIKV and RVFV; they have also been used in external quality assessment studies conducted by the European or international level [6,8,9,10,11].

The most important aspect was to compare the analytical sensitivity of the Lyoph-P&P assays against the results obtained when the primers and probe were prepared extemporaneously using the classic liquid format. For the two assays included in this study (CHIKV and RVFV), the analytical sensitivity of the Lyoph-P&P is not only equal to that observed with the liquid formulation, but even much better for CHIKV (0.56 copies/µL vs. 14 copies/µL), and slightly better for RVFV (more replicates detected for the last dilution providing positive results). The results observed with clinical samples tested for CHIKV RNA confirm the data obtained in analytical sensitivity studies. Detection of CHIKV RNA using Lyoph-P&P provides results in clinical samples that are at least equal and often better than those obtained with the extemporaneously prepared liquid formulation used as reference. Because of the low number of available clinical samples and due to the highly restrictive MicroOrganisms and Toxins (MOT) French regulation, it was not possible to perform the parallel study for RVFV. However, such comparative studies were done for a substantial number of assays that are routinely processed in the Clinical Microbiology Laboratory of the IHU Méditerranée Infection serving all beds of the Public Hospitals System of Marseille, France (Supplementary Table S1). Although this has to be addressed systematically when other assays will be transferred from the liquid formulation towards to Lyoph-P&P, these results are very promising and should engage in this direction for the detection of other pathogens.

Although all experiments described here were done using the Superscript^®^ III Platinium^®^ One-Step Quantitative RT-PCR kit (ThermoFischer), we have also used other enzymes such as the one-step qRT-PCR LightCycler^®^ Multiplex RNA Virus Master (Roche) that have provided similar results (data not shown).

As indicated in the Table 2, the concentration of primers and probe (to be lyophilized) had to be adjusted sometimes in order to obtain sensitivity comparable to that observed with extemporaneously prepared liquid preparation. The correction factor was determined empirically (1.25 and 1.50); interestingly, correction was not systematically necessary, and it could also be needed for one component of the reaction only, as shown with the RVFV assay.

It is important to underline that the rehydration of the Lyoph-P&P must be done as recommended in the protocol. Alternative protocols are likely to result in disappointing performances.

Despite different formats can be prepared as indicated in Table 3; Table 4, the question of the stability of Lyoph-P&P after rehydration is important to assess the versatility and flexibility of this solution. Indeed, at laboratory level, it is likely that one or two different formats (number of tests per vial) will be either prepared or ordered; as a consequence, the time during which rehydrated material can be stored without affecting the expected performances of the assay is a key factor. Interestingly, 7-day or 14-day storage at +4 °C had absolutely no deleterious effect on the performances; moreover, in some occurrences, sensitivity was even better after storage than after extemporaneous rehydration. Stability upon +4 °C storage after rehydration is important for the end-users because it prevents discarding reagents; this is not only important economically, but also renders the routine activity more comfortable when a large number of different pathogens are included in detection panels. The fact that rehydrated Lyoph-P&P was stable for at least 14 days after rehydration if stored at 4 °C is interesting because it allows to prepare or order vials containing greater number of tests without fearing the loss of material that is synonymous of increased costs. Stability of rehydrated material warrants versatility of the procedures, thus allowing to prepare/order vials containing 48- or 96-reactions. The same tendency was observed for the two assays suggesting that this phenomenon is not virus-dependent and may be expected with other detection assays.

Assessing the stability during shipping by maintaining the Lyoph-P&P at 37 °C up to 7 days intended to mimic degraded conditions potentially occurring during transportation at a given temperature, and also to address the possibility to perform shipping at ambient temperature. The excellent results observed at day 2 and day 4 support the possibility of ambient temperature shipping for this non-infectious material using rapid delivery companies such as WorldCourrier, UPS, DHL, FedEx, or similar ones that are capable to guarantee delivery within 4 days to almost any place in the world. Again, the promising results observed with CHIKV and RVFV must be confirmed for supplementary assays that will be developed. As examples of its versatility, the described procedure was used to prepare Lyoph-P&P with other RT-qPCR assays from the literature targeting Zika, dengue, and chikungunya viruses [12,13,14]; the corresponding Lyoph-P&P were shipped to overseas laboratories which were satisfied with the resulting performances on their own diagnostic platform (Thirion, unpublished data).

The opportunity to dispense with cold chain is also important to consider for economic reasons.

The last point to consider in the comparative analysis between liquid and Lyoph-P&P formulations deals with the interpretation of the PCR curves. The signal observed with low-copy samples close to the LOD, beyond Ct 35, is frequently weak as shown by low RFU level (Supplementary Data, dataset#1); interpretation of such results is frequently difficult and as a consequence often induces repeated testing for confirmation. The stronger the intensity of the signal, the easier the discrimination between clear positives and uncertain results. A detailed analysis denotes that for low copy samples, the intensity of the signals is clearly higher with Lyoph-P&P compared with the liquid formulation: approximately 200–1200 RFU vs. 2500–4900 RFU for RVFV, and approximately 120-470 RFU vs. 430-790 RFU for CHIKV (Suppl Data, dataset#1).

Recently, increased robustness of real-time PCR assays has been achieved by combining two targets in a unique reaction tube in order to prevent false negative results that may arise from point mutations/deletions/insertions frequently observed with emerging pathogens, even more frequently with pathogens with RNA genome [14,15,16,17]. This tendency implies the need to increase the number of different primers and probe within a single assay, which renders the preparation of the reaction mix even more prone to human errors. Whether or not this tendency should expand, Lyoph-P&P would be even more attracting for diagnostic activities in routine clinical microbiology laboratories. The recipient laboratory will have to perform minimal validation steps before the Lyoph-P&P can be included in the routine diagnostic activity.

In conclusions, the advantages of Lyoph-P&P reside (i) in its stability for shipping and storage, (ii) in the drastically reduced number of manipulations to prepare the ultimate reaction tube/plate to be placed in the thermocycler, (iii) in its flexibility in terms of number of reactions per prepared vial (1 to 96, even 1 to 384). Utilization of Lyoph-P&P is an easy manner to transfer diagnostic capacity between laboratories.

## Figures and Tables

**Table 1:** Oligonucleotide primers and probes used in this study.

Primer/Probe	Sequence (5′-3′) ^a^	Target	Position	Amplicon (nts)	[nM]	Reference
F-CHIK (forward)	AAGCTYCGCGTCCTTTACCAAG	E1	10,380–10,401	208	900	
R-CHIK (reverse)	CCAAATTGTCCYGGTCTTCCT	E1	10,568–10,588	208	900	[6]
P-CHIK (probe)	FAM^b^-CCAATGTCYTCMGCCTGGACACCTTT-TAMRA	E1	10,479–10,504	208	200	
RVS (forward)	AAAGGAACAATGGACTCTGGTCA	G2	349–371	94	1000	[2]
RVAs (reverse)	CACTTCTTACTACCATGTCCTCCAAT	G2	443–417	94	1000	
RVP (probe)	FAM-AAAGCTTTGATATCTCTCAGTGCCCCAA-TAMRA	G2	388–416	94	200	

^a^ IUPAC codes used to indicate degenerate positions; ^b^ FAM: 6-carboxyfluorescein reporter dye.

**Table 2:** Primers and Probe mix solutions.

				nb Tests/Vial				nb Tests/Vial	
				8*	16*		24*	48*	96*
**CHIKV**	**nM/rxn**	**Lyophilization Factor**	**Volume (µL) *qs* 30 µL**	**Volume (µL)**				**Volume (µL)**	
F-CHIK 10µM	900	1.25	3.4	30.4	60.8	F-CHIK 100µM	8.8	17.9	35.1
R-CHIK 10µM	900	1.25	3.4	30.4	60.8	R-CHIK 100µM	8.8	17.9	35.1
P-CHIK 10µM	200	1.25	0.75	6.8	13.5	P-CHIK 100µM	1.9	4.0	7.8
Dye+Sucrose (2 µL)			2**	18	36	Dye+Sucrose (1µL)	26	53	104
MGW				114.5	29	MGW	154.5	107.3	18
**RVFV**									
RVS 10µM	1000	1	3	27	54	RVS 100µM	7.8	15.9	31.2
RVAs 10µM	1000	1	3	27	54	RVAs 100µM	7.8	15.9	31.2
RVP 10µM	200	1.25	0.75	6.8	13.5	RVP 100µM	1.9	4.0	7.8
Dye+Sucrose (2 µL)				18	36	Dye+Sucrose (1µL)	26	53	104
MGW				121.3	42.5	MGW	156.5	111.3	25.8

MGW, Molecular Grade Water; rxn, reaction. * safety margin was included in the calculation: to prepare a eight-test vial, volumes are calculated for nine tests; for 16-test vial, volumes of 18 tests; for 24-test vial, volumes of 26 tests; for 24-test vial, volumes of 26 tests; for 48-test vial, volumes of 53 tests; for 96-test vial, volumes of 104 tests. ** when the number of tests/vial is ≥the volume of (dye+sucrose) mix added per vial is 1 uL/test.

**Table 3:** Vial regeneration according to the number of tests per vial.

Number of Tests/Vial (*)	8 (+1)	16 (+2)	24 (+2)	48 (+5)	96 (+8)
AE Elution buffer (µL)	39.6	79.2	114.4	233.2	457.6

(*), supplementary test planned to compensate for possible pipetting errors.

**Table 4:** PCR Mix preparation when using the Superscript^®^ III Platinium^®^**.**

Number of Tests	1	8(+1)	16(+2)	24(+2)	48(+5)	96(+8)
SSIII PCR Mix (µL)	15	135	270	390	795	1560
SSIII Enzyme (µL)	0,6	5.4	10.8	15.6	31.8	62.4
rehydrated P&P (µL)	4.4	39.6	79.2	114.4	233.2	457.6
Total Volume (µL)	20	180	360	520	1060	2080

**Table 5:** Stability at 4 °C after rehydration of Lyoph-P&P compared with liquid format.

	**CHIKV Lyoph-P&P**						**CHIKV Liquid**	
	**Ct day 0**		**Ct day 7**		**Ct day 14**		**Ct day 0**	
**RNA Copies/µL**	**Mean** **^a^**	**SD** **^b^**	**Mean**	**SD**	**Mean**	**SD**	**Mean**	**SD**
8800	28.1	0.5	27.63	0.1	**27.43**	0.3	28.72	0.1
880	**31.7**	0.2	31.79	0.2	31.91	0.1	32.98	0.31
88	35.4	0.5	**35.21**	0.6	36.36	0.6	36.58	0.53
	**RVFV Lyoph-P&P**						**RVFV Liquid**	
	**Ct day 0**		**Ct day 7**		**Ct day 14**		**Ct day 0**	
**RNA Copies/µL**	**Mean**	**SD**	**Mean**	**SD**	**Mean**	**SD**	**Mean**	**SD**
7700	26.93	0.06	30.46	0.26	30.01	0.14	**29.48**	0.89
770	**30.73**	0.33	33.69	0.16	34.11	0.09	32.97	0.19
77	**34.52**	0.06	36.43	0.29	37.32	0.66	37.25	0.06

^a^ mean of three replicates; the lowest Ct values for each concentration are in bold; ^b^ standard deviation.

**Table 6:** Stability of Lyoph-P&P at 37 °C for shipping mimicry.

	CHIKV Lyoph-P&P								RVFV Lyoph-P&P								
	Ct day 0		Ct day 2		Ct day 4		Ct day 7			Ct day 0		Ct day 2		Ct day 4		Ct day 7	
RNA Copies/µL	Mean	SD	Mean	SD	Mean	SD	Mean	SD	RNA Copies/µL	Mean	SD	Mean	SD	Mean	SD	Mean	SD
8800	27.26	0.96	25.82	0.05	25.21	0.18	**24.98**	0.22	7700	**26.87**	0.16	27.18	0.08	28.09	0.18	30.57	0.05
880	31.93	0.30	29.16	0.15	29.51	0.20	**28.64**	0.33	770	30.88	0.06	**30.33**	0.05	30.90	0.12	33.61	0.22
88	35.86	2.04	33.42	1.71	33.04	0.51	**31.34**	1.44	77	34.52	0.18	**33.88**	0.14	34.42	0.20	36.10	0.47

The lowest Ct values for each concentration are bolded.

**Table 7:** Analytical sensitivity: comparison between Lyoph-P&P and extemporaneously prepared liquid formulation.

**CHIKV**		**Lyoph-P&P**		**Liquid**	
**Dilution of RNA**	**RNA Copies/µL**	**Detected/Tested**	**Ct, Mean (SD)**	**Detected/Tested**	**Ct, Mean (SD)**
1.00 × 10^−8^	8800	3/3	27.26 (0.04)	3/3	27.77 (0.43)
8.00 × 10^−11^	70	3/3	30.64 (0.12)	3/3	35.05 (0.38)
1.60 × 10^−11^	14	3/3	32.87 (0.15)	3/3	39.68 (0.52)
3.20 × 10^−12^	3	3/3	35.62 (0.43)	0/3	>40
6.40 × 10^−13^	0.56	3/3	36.43 (0.40)	0/3	>40
1.28 × 10^−13^	-	0/3	>40	0/3	>40
**RVFV**		**Lyoph-P&P**		**Liquid**	
**Dilution of RNA**	**RNA Copies/µL**	**Detected/Tested**	**Ct, Mean (SD)**	**Detected/Tested**	**Ct, Mean (SD)**
1.00 × 10^−8^	7700	3/3	30.14 (0.14)	3/3	30.73 (0.16)
8.00 × 10^−11^	62	3/3	34.93 (0.24)	3/3	36.93 (0.49)
1.60 × 10^−11^	12	3/3	36.91 (0.31)	3/3	39.23 (0.15)
3.20 × 10^−12^	2	3/3	38.30 (1.34)	1/3	39.76
6.40 × 10^−13^	-	0/3	>40	0/3	>40
1.28 × 10^−13^	-	0/3	>40	0/3	>40

**Table 8:** Sensitivity of Lyoph-P&P and extemporaneously prepared liquid formulation for CHIKV RNA detection.

	Liquid		Lyoph-P&P			Liquid		Lyoph-P&P	
Sample ID	Ct, Mean ^a^	SD	Ct, Mean	SD	Sample ID	Ct, Mean	SD	Ct, Mean	SD
20281	21.33	0.36	**20.58**	**0.09**	20591	19.67	0.06	**19.58**	**0.03**
20296	21.02	0.23	**20.28**	**0.09**	20594	16.34	0.13	**15.87**	**0.11**
20297	22.50	0.08	**22.14**	0.20	20621	18.32	0.29	**18.17**	**0.14**
20299	20.26	0.28	**20.21**	**0.17**	20625	18.97	0.16	**18.89**	**0.04**
20355	20.22	0.24	**20.09**	**0.19**	20631	16.35	0.16	**15.92**	**0.02**
20359	16.80	0.08	**16.34**	**0.02**	20632	22.87	0.24	**22.48**	**0.04**
20361	20.38	0.25	**20.06**	**0.21**	20634	28.16	0.23	**27.74**	**0.13**
20369	22.16	0.24	**22.04**	**0.08**	20636	19.29	0.12	**19.03**	0.15
20381	20.79	0.08	**20.49**	0.18	20637	23.45	0.11	**23.23**	**0.10**
20391	19.48	0.14	**19.11**	**0.06**	20639	16.61	0.16	**16.42**	**0.11**
20396	19.53	0.09	**19.43**	0.09	20660	20.49	0.07	**20.26**	**0.03**
20399	**17.50**	0.23	17.52	**0.08**	20677	18.23	0.23	**18.00**	**0.03**
20400	23.12	0.04	**22.74**	0.07	20679	20.98	0.04	**20.91**	0.12
20407	28.19	0.25	**27.51**	**0.15**	20682	**18.31**	0.03	18.48	0.17
20409	21.17	0.31	**20.91**	**0.12**	20683	25.41	0.07	**25.32**	0.14
20461	19.34	0.09	**19.00**	0.12	20684	20.48	0.02	**20.41**	0.11
20468	23.24	0.11	**23.05**	**0.03**	20691	27.28	0.13	**27.19**	**0.03**
20481	22.96	0.10	**22.61**	**0.05**	20692	19.23	0.13	**19.10**	0.18
20482	26.30	0.14	**26.18**	0.21	20695	16.56	0.19	**16.39**	**0.13**
20485	16.49	0.35	**16.15**	**0.11**	20696	21.00	0.19	**20.65**	**0.04**
20506	19.14	0.10	**18.96**	0.29	20814	22.84	0.14	**22.50**	**0.06**
20507	22.11	0.08	**21.91**	0.10	20827	19.25	0.05	**19.04**	0.16
20516	18.50	0.03	**18.51**	0.09	20832	20.82	0.17	**20.41**	**0.05**
20519	16.30	0.12	**16.37**	**0.04**	20880	18.39	0.03	**18.07**	0.08
20522	17.32	0.05	**17.43**	0.07	20909	17.74	0.08	**17.32**	**0.04**
20524	18.74	0.10	**18.92**	**0.09**	20911	18.16	0.02	**17.97**	0.08
20537	23.13	0.18	**23.00**	**0.13**	20912	23.84	0.12	**23.47**	**0.05**
20566	18.60	0.05	**18.41**	0.08	20916	19.62	0.05	**19.50**	0.06
20562	19.17	0.05	**18.93**	0.25	20875	21.59	0.04	**21.41**	0.06
20563	20.00	0.23	**19.71**	**0.19**	20756	**20.46**	0.05	20.51	0.07
20607	19.14	0.17	**19.01**	**0.10**	20899	17.39	0.05	**17.34**	0.06
20610	**21.54**	0.12	21.55	0.49	20900	22.69	0.01	**22.57**	0.08
20612	26.29	0.23	**25.94**	**0.21**	20925	18.24	0.07	**18.18**	0.10
20613	25.94	0.27	**25.83**	**0.25**	20856	21.26	0.06	**21.16**	0.06
20614	**22.10**	0.29	22.27	**0.16**	*IVT*	25.13	0.43	**25.00**	**0.34**
20616	20.24	0.12	**20.15**	0.18	Negatives ^b^	-		-	

^a^ mean of three replicates for plasma samples and five replicated in vitro transcribed RNA; the lowest Ct values and SD are in bold; ^b^ 10 samples tested; IVT, in vitro transcribed RNA.

## Supplementary Materials

Supplementary materials can be found at https://www.mdpi.com/1999-4915/12/2/159/s1.
