# Supplementary material for: Lyophilized Matrix Containing Ready-to-Use Primers and Probe Solution for Standardization of Real-Time PCR and RT-qPCR Diagnostics in Virology
Source: Viruses. 2020 Jan 30;12(2):159. doi: 10.3390/v12020159 (PMC7077261; doi:10.3390/v12020159)
Supplement: Supplementary file 1 [file viruses-12-00159-s001.pdf]

Arboviruses:

Phlebovirus genus

- Toscana virus
- Sandfly fever Naples phlebovirus
- Sandfly fever Sicilian phlebovirus
- Severe fever with thrombocytopenia syndrome phlebovirus (SFTSV)
- Rift Valley fever phlebovirus

Flavivirus genus

- Dengue 1 virus
- Dengue 2 virus
- Dengue 3 virus
- Japanese encephalitis virus
- West Nile virus
- Yellow fever virus
- Spondweni virus
- Zika virus
- Tick-borne encephalitis virus
- Usutu virus

Alphavirus genus

- Mayaro virus
- O'Nyong nyong virus

Orthobunyavirus genus

- Oropouche bunyavirus

Orthonairovirus genus

- Crimean–Congo hemorrhagic fever nairovirus

DNA viruses

Herpesviridae

- Varicella-zoster virus
- Herpes simplex 1 &-2
- Human Herpes virus 6, 7, 8
- Cytomegalovirus
- Epstein–Barr virus

Poxviridae

- Vaccinia virus
- Cowpox virus
- Monkeypox virus
- Orf virus
- Pseudocowpox virus
- Bovine papular stomatitis virus

Other

- Adenoviruses
- Poliomavirus JC & BK
- Parvovirus B19

Others viruses :

- Measles virus
- Mumps virus
- Rubella virus
- Norovirus GG1 & GG2
- Sapovirus
- Coronavirus 229E, OC43, NL63, HKU1
- Parechovirus
- Human respiratory syncytial virus
- Influenza A virus, B virus, C virus
- Human parainfluenza virus 1,2,3,4
- Enterovirus
- Rabies virus
- Ebola virus

Suppl. Table. List of viruses fro which primers and probe mix have been prepared in the form of Lyoph-P&P and which are routinely used for diagnosis in the Clinical Microbiology Laboratory of the IHU Méditerranée Infection serving the whole Public Hospital System of Marseille, France.
